# Rare pathogenic mutation of *KCNH2* p.D501N associated with early-onset malignant long QT syndrome

**DOI:** 10.3389/fcvm.2026.1592799

**Published:** 2026-06-10

**Authors:** Yubi Lin, Xingchen Li, Mingsui Gao, Yechang Chen, Zhuguo Wu, Jia Chen

**Affiliations:** 1 The Dongguan TaiXin Hospital, Affiliated to Guangdong Medical University, Dongguan, China; 2 The Second Department of Cardiology, The Affiliated Guangdong Second Provincial General Hospital of Jinan University, Guangzhou, China; 3 The First Dongguan Affiliated Hospital, Guangdong Medical University, Dongguan, China

**Keywords:** KCNH2, long QT syndrome, ventricular arrhythmia, voltage-sensing domain, whole-exome sequencing

## Abstract

**Introduction:**

Long QT syndrome (LQTS) is a life-threatening inherited channelopathy that is prone to triggering torsades de pointes (TdP), ventricular tachycardia (VT), ventricular fibrillation (VF), and sudden cardiac death.

**Materials and methods:**

Whole-exome sequencing (WES) was performed on a proband with LQTS, and candidate variants were validated by Sanger sequencing. Bioinformatics methods were used to analyze the pathogenicity of the variants.

**Results:**

A proband who was a 16-year-old girl presented with a prolonged QT interval, TdP, VT, and VF. She carried the *de novo KCNH2* p.D501N mutation, which is localized to the S3 helix of the voltage-sensing domain of Kv11.1 and predicted to be “deleterious” by the SIFT, PolyPhen-2, and MetaSVM algorithms. Secondary structure prediction showed that this mutation increases the proportion of alpha helices and extended strands, while decreasing the proportion of the random coil. In addition, this substitution induces buried charge replacement, breakage of the buried salt bridge formed by p.D501 and p.R534, and cavity expansion (with p.R537 moving markedly outward). It also leads to abnormal changes in the interactions among p.R537, p.R534, p.Y493, p.V533, and p.W497, resulting in spatial structure displacement and deflection. Compared with wild-type *KCNH2*, *KCNH2* p.D501N exhibits decreased molecular weight, total number of negatively charged residues (Arg + Lys), oxygen content, charged residues (B: Asx, D: Asp, E: Glu, H: His, K: Lys, R: Arg, Z: Glx), and acidic residues (B + D + E + Z), while it shows increased theoretical isoelectric point (PI), hydrogen and nitrogen content, isoelectric point, net charge, and reduced probability of the formation of inclusion bodies.

**Conclusions:**

The *KCNH2* p.D501N mutation significantly alters the secondary/tertiary structure and physicochemical properties of the K_v_11.1 protein, which may impair the cardiac I_Kr_ current and ultimately induce early-onset malignant LQT2 in a Chinese family. This condition requires ICD therapy combined with long-term β-blocker administration and potassium–magnesium supplementation.

## What is new?

- We report the case of a 16-year-old Chinese girl who carried the *de novo KCNH2* p.D501N mutation and presented with a QT interval ≥600 ms, TdP, VT, and VF without any structural cardiac disease.
- The mutation is predicted to alter the K_v_11.1 secondary structure (↑*α*-helices/extended strands, ↓random coil) and induce voltage sensor domain (VSD) S3-S4 tertiary abnormalities (buried charge replacement, D501-R534 salt bridge breakage, 458.352 Å^3^ cavity expansion).
- *KCNH2* p.D501N causes early-onset malignant LQT2 requiring ICD therapy with long-term β-blocker and potassium–magnesium supplementation.

## Introduction

Cardiac channelopathies commonly occur in young patients without apparent structural heart disease who are usually unaware of their cardiac disorder, and sudden cardiac death (SCD) may be the first presenting symptom for them. The most common cardiac channelopathy is long QT syndrome (LQTS), which can be either congenital or acquired. Congenital LQTS is considered to be rare, with a prevalence of 1/2500 in the general population (1). LQTS is commonly associated with life-threatening arrhythmia, including torsade de pointes (TdP), ventricular tachycardia (VT), ventricular fibrillation (VF), and even ventricular electrical storm, which leads to syncope, seizures, SCD, and unexplained sudden death (USD) (2). LQTS is largely an autosomal-dominant disorder, with approximately 80% of cases caused by pathogenic mutations in three ion channel genes (LQT1, *KCNQ1*-encoded K_v_7.1; LQT2, *KCNH2*-encoded K_v_11.1; and LQT3, SCN5A-encoded Na_v_1.5). *KCNH2* on chromosome 7q36 encodes the K_v_11.1 voltage–gated potassium channel alpha subunit, which is responsible for the delayed rectifying potassium current (I_Kr_). I_Kr_ is a major determinant of cardiac action potential duration in cardiomyocytes and the heart. *KCNH2* abnormalities, especially loss-of-function mutations, increase the transmural dispersion of repolarization and eventually induce QT interval prolongation, a notched T wave in electrocardiogram, re-entry tachyarrhythmia, and TdP, which were classified as Type 2 of LQTS (LQT2) by researchers (3, 4). In this study, we report the case of a patient with early-onset malignant LQT2 who carried a *de novo KCNH2* p.D501N mutation in a Chinese family. We verified the mutation by whole-exome sequencing (WES), elucidated the detailed molecular pathogenic mechanism of the mutation through a bioinformatics analysis, and provided an 11-year long-term clinical follow-up data.

## Materials and methods

### Ethical compliance

All study participants signed the informed consent form. All procedures performed in the study involving human participants followed the Declaration of Helsinki and ethical standards approved by the Guangdong Medical Institutional Review Board and Medical Ethics Committees [No. GDREC2016001H (R1)]. Detailed clinical information was collected from the participants. This information pertained to their family history, age of presentation, initial symptoms of VT, physical examination, and electrocardiograms (ECGs) and echocardiogram based on their informed consent. The clinical diagnostic criteria for LQTS depended mainly on the clinical standard score (5, 6).

### Whole-exome sequencing

In the family of the patient in this study, proband (II: 1) was diagnosed with LQTS and thus selected to perform WES. Genomic DNA samples of patients were isolated from peripheral blood using a standard DNA extraction protocol. The isolated genomic DNA was then fragmented into 150–200 bp and subjected to the process of DNA library preparation using established Illumina paired-end protocols. Adaptor-ligated libraries were amplified via PCR. A portion of each library was used to create an equimolar pool. Each pool was amplified to enrich targets sequenced by the Agilent SureSelectXT Target Enrichment System (Agilent Technologies Inc., Santa Clara, CA, USA). According to the manufacturer's protocol, whole-exome capture was performed using the Agilent SureSelectXT Human All Exon 50 Mb Kit (Agilent Technologies Inc.). According to the manufacturer's instructions, the exome-enriched libraries were sequenced using the Illumina Hiseq 2000 platform (Illumina, San Diego, CA, USA), and 100 bp paired-end sequencing reads were generated. Each sample was sequenced per lane to obtain an average theoretical depth of 100× (7).

### Read mapping, variant detection, and functional annotation

Raw reads were collected for quality control, in which low-quality reads were filtered, and 3′/5′ adapters were trimmed using the Trim Galore program. Clean reads were aligned to the human reference genome (University of California Santa Cruz, UCSC build hg19) using the Burrows–Wheeler Aligner (BWA) program. The quality scores were recalibrated, and the reads were realigned to the reference genome using the Genome Analysis Toolkit (GATK) software package. After duplicate reads were excluded, insertions–deletions (indels) and single-nucleotide polymorphisms (SNPs) were called using the GATK or Sequence Alignment/Map tools (SAM tools) (7).

The filtering criteria were applied as follows: (1) Only missense, nonsense, coding insertions/deletions (indels), and splice-site variants were retained; (2) Variants with a minor allele frequency (MAF) >1% in the 1000 Genomes Project (1000G; https://www.internationalgenome.org/) and Exome Aggregation Consortium (ExAC; http://exac.broadinstitute.org) databases were excluded; (3) Only variants located in susceptibility genes associated with hereditary arrhythmias and cardiomyopathies were kept. Potentially pathogenic variants were validated in other family members by Sanger sequencing. Detailed methods have been described in our previous studies (8, 9). The remaining variants were categorized as “pathogenic (P),” “likely pathogenic (LP),” “variant of uncertain significance (VUS),” “likely benign (LB),” or “benign (B)” based on the ClinVar database. In addition, in silico functional prediction of single-nucleotide variants (SNVs) was performed using the SIFT (http://sift.jcvi.org/www/), PolyPhen-2 (Polymorphism Phenotyping v2; http://genetics.bwh.harvard.edu/pph2/), MetaSVM, and CADD algorithms (10). Variants with a CADD score <20 were excluded, as higher CADD scores indicate a greater likelihood of deleterious effects: a scaled score ≥10 denotes a raw score in the top 10% of all possible reference genome SNVs, while a score ≥20 indicates a raw score in the top 1%. Synonymous variants and those with a CADD score <20 were also excluded, unless they were classified as “pathogenic” or “likely pathogenic” in ClinVar (11). The pathogenicity of the coding sequence variants was further evaluated using InterVar, in accordance with the criteria established by the American College of Medical Genetics and Genomics (ACMG) (12). Finally, cosegregation of candidate variants within the family was assessed using Sanger sequencing.

### Sanger sequencing

When the suspected pathogenic mutations were obtained in each step, they were screened again using Sanger sequencing in the other members of the family. The primers designed with Primer Premier 5.0 were used (7).

The primers of *KCNH2* p.D501N in Sanger sequencing were as follows: forward primer: ATGTCATCGCTCCTGCCCC; reverse primer:CCTCCACCCCACTACCTCCC. We conducted Sanger sequencing not only in the family member I:1 and I:2 but also in the proband of II:1.

### Conservation analyses

We used the UCSC Genome Browser (Human hg19 assembly) algorithms of “Vertebrate Multiz Alignment & Conservation (100 Species)” for conservation analysis. Multiple alignments were generated using Multiz tool in the UCSC/Penn State Bioinformatics comparative genomics alignment pipeline. The resulting best-in-genome pairwise alignments were progressively aligned using Multiz/AutoMZ, following the tree topology diagrammed above, to produce multiple alignments (13).

### SOPMA secondary structure prediction

We performed secondary structure prediction using the self-optimized prediction method with alignment (SOPMA) tool. SOPMA is an improvement of the SOPM. Improvement to this method was done because SOPMA takes into account information from an alignment of sequences belonging to the same family. If there are no homologous sequences, SOPMA prediction turns out to be the same as that of the SOPM (14). It predicts the secondary structure (*α*-helix, extended strand, random coil) by integrating evolutionary conservation information from homologous sequences, with the strength of high consistency for conserved regions but limited accuracy for proteins with few homologs.

### Protein physical–chemical prediction

In this study, we used the ProtParam, EMBOSS Pepstats, ProtScale, and NetPhos algorithms for analyzing physical–chemical properties, hydrophobicity, transmembrane domain, and phosphorylation (15–18). ProtParam and EMBOSS Pepstats calculate properties such as molecular weight, isoelectric point, and charge distribution based on amino acid composition; their strengths are achieving comprehensive parameters while maintaining fast computation, whereas their limitations include inability to reflect conformation-dependent dynamic properties. ProtScale evaluates hydrophobicity using predefined amino acid scales, and NetPhos predicts phosphorylation sites via machine learning trained on experimental data; both are high-throughput algorithms but ignore spatial structure effects on residue accessibility.

### Missense3D prediction

Missense3D (http://missense3d.bc.ic.ac.uk/missense3d/) predicts the structural changes introduced by an amino acid substitution and is suitable for analyzing both PDB coordinates and homology-predicted structures (19). The input requires the protein UniProt ID, amino acid position, substitution, and the PDB ID of the experimental 3D structure. A link to the available PDB structures is provided. It identifies structural abnormalities (e.g., salt bridge breakage and cavity expansion) by superimposing mutant and wild-type 3D models, with the strength of intuitive visualization of mutation-induced structural alterations, but its accuracy relies on the quality of the input 3D structure (experimental or homologous model).

## Results

### Clinical presentation and familial characteristics

**Figure 1:**
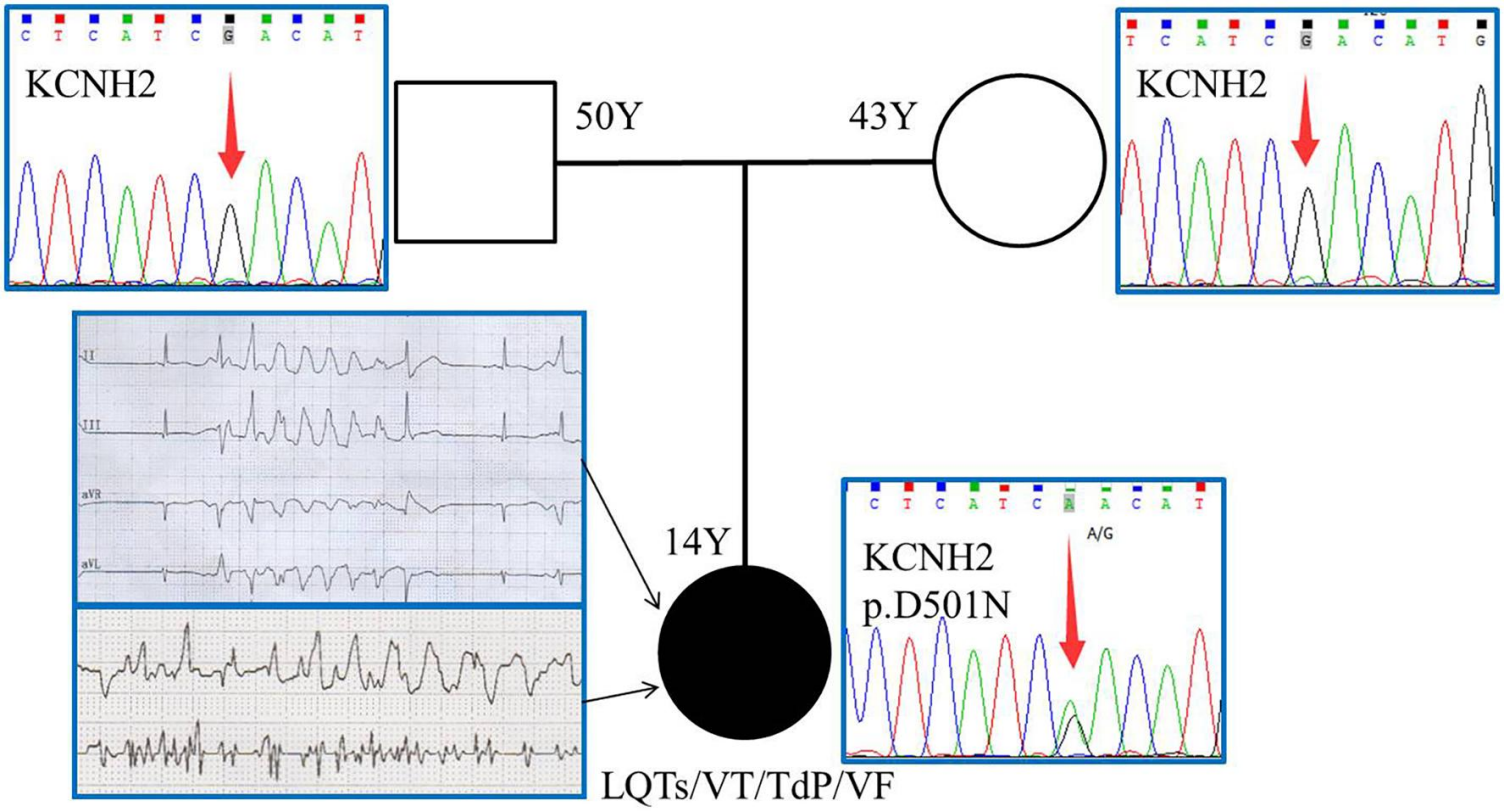
Familial pedigree of the proband with *KCNH2* p.D501N. This figure depicts the pedigree structure of the family involved in the study. The proband (II:1) is a 16-year-old girl diagnosed with early-onset malignant LQT2, who presented with recurrent syncope, torsades de pointes (TdP), ventricular tachycardia (VT), and ventricular fibrillation (VF), which required implantable cardioverter-defibrillator (ICD) implantation. Her father (I:1) and mother (I:2) had no clinical manifestations related to LQTS (e.g., syncope, palpitations, sudden cardiac death) and showed no abnormalities in electrocardiography (ECG) and echocardiography. A genetic testing confirmed that only the proband carried the heterozygous *KCNH2* p.D501N mutation, while the parents did not, indicating that this is a *de novo* mutation. Symbols: □ = male; ○ = female; ● = affected female and disease (proband); arrow = proband.

**Figure 2:**
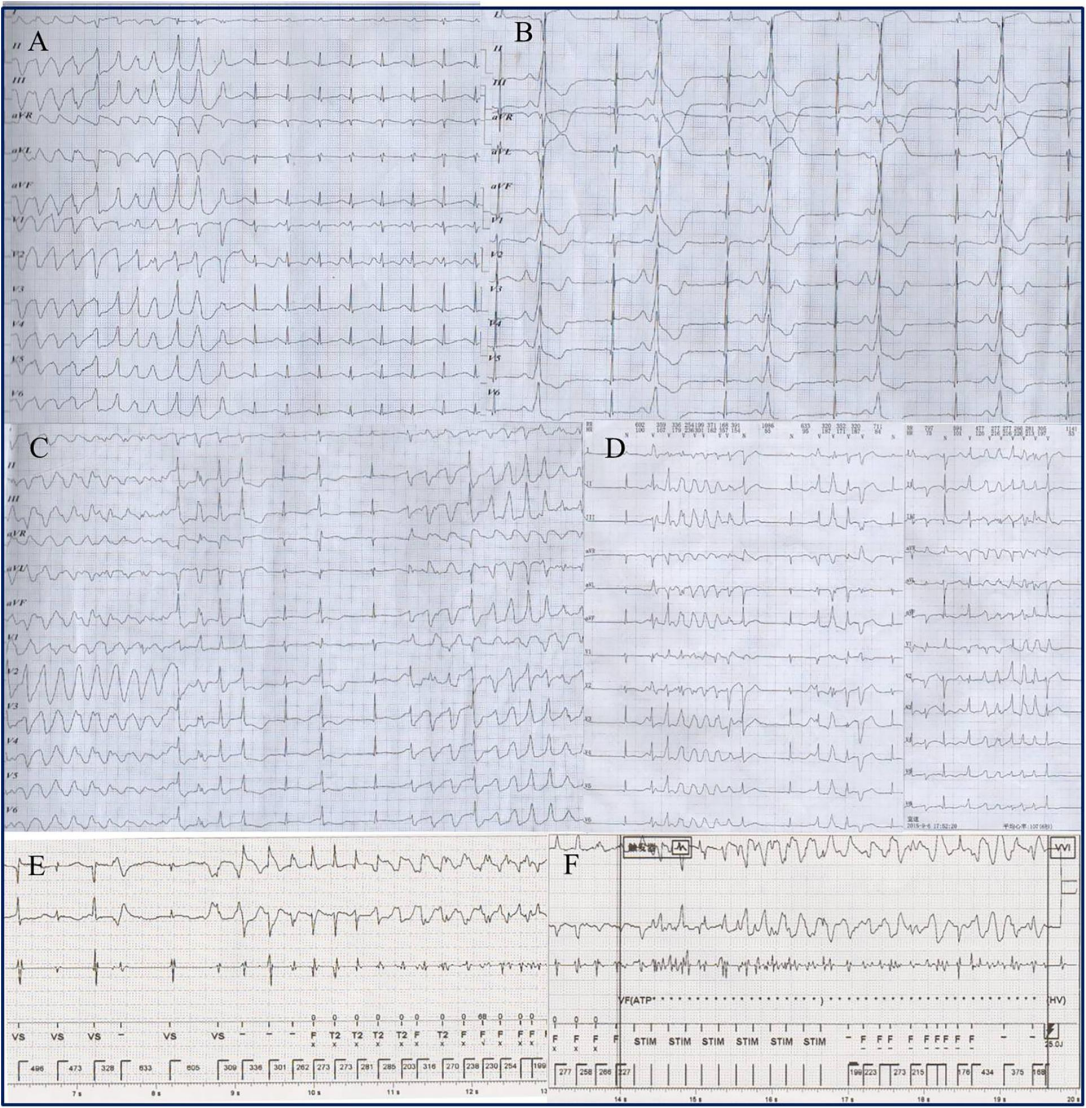
Ambulatory eAmbulatory electrocardiographic and intracardiac electrogram characteristics of the proband. Ambulatory electrocardiogram tracings show an episode of torsade de pointes (TdP) (**A**), frequent ventricular premature beats in bigeminy originating from the left ventricular outflow tract (**B**), rhythm progression from ventricular tachycardia/ventricular fibrillation to non-sustained ventricular tachycardia followed by restoration of sinus rhythm and recurrent torsade de pointes (**C**), and frequent multifocal non-sustained ventricular tachycardia (**D**). Intracardiac electrogram tracings from the ICD demonstrate frequent premature ventricular beats progressing to ventricular tachycardia and ventricular fibrillation, which are successfully terminated by ICD shocks (**E,F**).

A 16-year-old girl with “recurrent syncope for two years” as the main complaint was hospitalized in September 2015 (Figure 1). She had been experiencing syncope since 2013. When she presented to the hospital two years later, the condition lasted for 4–5 min, complicated by a loss of consciousness. After she woke up, she felt dizzy, palpitation, and limb weakness, but she had no chest pain, nausea, and vomiting. During the 2-year period, paroxysmal syncope occurred more than 10 times. As shown in Figure 2, the admission electrocardiogram revealed that the QT interval (≥600 ms) was obviously and aberrantly prolonged, accompanied by frequent ventricular premature beats originating from the left ventricular outflow tract and characterized by an R-on-T pattern. II:1 presented with recurrent TdPs, frequent ventricular premature beats, VTs, and VFs (Figures 2A–D). According to the diagnosis criteria of LQTS, the clinical standard score of the proband was six points according to the guidelines. Subsequently, II: 1 was implanted using an intracardiac implantable defibrillator (ICD). The occurrences of TdP and VF were recorded by ICD monitoring, and the patient required intracardiac defibrillation therapy (Figures 2E,F). The results of her cardiac magnetic resonance imaging, echocardiography, chest radiographs, and thyroid examination demonstrated no significant abnormalities. Neither family members I:1 and I:2 presented any clinical manifestations related to LQTS (e.g., syncope, palpitations, seizures, or unexplained cardiac events). Comprehensive clinical evaluations, including ECG and echocardiography, were performed for both parents, and the results showed no QT interval prolongation, ventricular arrhythmia, or structural heart disease.

After ICD implantation in 2015, the patient was initiated on long-term medication therapy with metoprolol succinate sustained-release tablets (47.5 mg, oral, once daily) and potassium aspartate and magnesium aspartate tablets (one tablet per dose, oral, three times daily). A telephonic follow-up conducted on 9 February 2026, confirmed good medication adherence. During this 11-year follow-up period (2015–2026), the therapeutic regimen achieved significant efficacy: recurrent syncope was completely resolved; the frequency of TdP/VT episodes was reduced, with ICD discharges recorded only twice in 2024 (last discharge on October 2024). The ICD generator was changed in November 2024 because of battery depletion, and no further arrhythmic events requiring ICD intervention were reported by February 2026. The patient had no adverse drug reactions such as bradycardia, hypotension, or electrolyte disturbance during medication use.

### Cosegregation analysis of clinical phenotype and genotype

**Figure 3:**
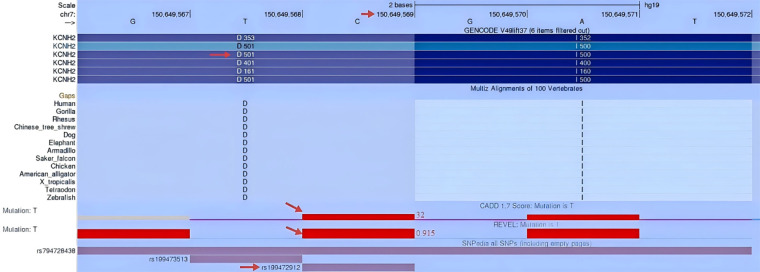
A conservation analysis of the *KCNH2* p.D501 residue across 100 vertebrate species. This figure illustrates the evolutionary conservation of the amino acid residue at position 501 of the *KCNH2*-encoded K_v_11.1 protein, analyzed using the “Vertebrate Multiz Alignment and Conservation (100 Species)” tool in the UCSC Genome Browser. (1) A schematic diagram of *KCNH2* protein transcripts: The D501 residue is conserved across different transcripts (e.g., D161 in short transcript, D405 in intermediate transcript, and D501 in full-length transcript), indicating its functional importance. (2) Cross-species alignment results: The D501 residue (marked with a red star) is highly conserved among 100 vertebrate species, including primates (e.g., Gorilla, Rhesus), mammals (e.g., Elephant, Dog), birds (e.g., Saker falcon, Chicken), reptiles (e.g., American alligator), amphibians (e.g., X. tropicalis), and fish (e.g., Zebrafish, Tetraodon). All species retain an aspartic acid (D) at this position, except for the proband's mutation (D→N). Functional prediction tools (CADD score = 32, REVEL score = 0.915, red bars) consistently classified the p.D501N variant as deleterious, strongly supporting its pathogenic role in the development of long QT syndrome.

In the family of this patient (Figure 1; Table 1), a set of candidate genes associated with cardiomyopathies and arrhythmias were screened using the WES data of II: 1. The results showed that *KCNH2* p.D501N carried by II: 1 was associated with LQT2. Based on the American College of Medical Genetics and Genomics (ACMG) criteria, the pathogenicity of *KCNH2* p.D501N was classified as “uncertain significance (PM2, PP3, PP5).” The *KCNH2* p.D501N mutation carried by II:1 was predicted to be pathogenic by six commonly used bioinformatics tools. Among them, the SIFT, Polyphen2, and MetaSVM algorithms predicted the mutation as “deleterious”; the CADD prediction score (Figure 3) was >20 (indicating the top 1% harmful variants in the genome). According to variantbrowser.org/KCNH2/variantinfo/c.1501G>A, the PROVEAN score was −4.633 (≤−2, indicating pathogenicity), while the REVEL score was 0.915 (≥0.75, indicating likely pathogenicity). The consistent predictions of different tools verify the reliability of the pathogenicity prediction of this mutation. The penetrance of LQT2 for *KCNH2* p.D501N was estimated at 38%. According to the Clinvar database, *KCNH2* p.D501N (rs199472912) has been reported in the literature in one individual affected with LQT2. Only II: 1 carried the heterozygous mutation of *KCNH2* p.D501N, while the girl’s father (I:1) and mother (I:2) did not. Therefore, *KCNH2* p.D501N was a *de novo* mutation in the proband of II:1.

**Table 1:** The potential pathogenic variants of the proband.

Chr	Start	Ref	Alt	Gene	Amino acid Change	1,000 g	SNP code	SIFT	Polyphen	MetaSVM	Clinvar	OMIM disease	Pathogenicity by ACMG criteria
chr1	228432177	G	A	*OBSCN*	NM_052843:exon11:c.G3386A:p.R1129Q	–	–	T(0.34)	D(0.93)	T(−0.61)	–	–	US
chr12	2695057	C	A	*CACNA1C*	NM_000719:exon18:c.C2517A:p.N839K	–	–	T(0.84)	B(0.17)	D(0.05)	–	Brs	US
chr7	150649569	C	T	*KCNH2*	NM_172056:exon6:c.G1501A:p.D501N	–	rs199472912	D(0)	D(1.00)	D(0.91)	P	LQTs	US
chr9	139390546	G	A	*NOTCH1*	NM_017617:exon34:c.C7645T:p.R2549C	0.0006	rs200893930	D(0.05)	B(0.33)	T(−0.21)	–	–	US
chrX	108868150	G	A	*KCNE5*	NM_012282:exon1:c.C100T:p.R34C	–	rs377062108	D(0.04)	B(0.22)	T(−0.97)	–	–	US

Chr, chromosome; Ref, reference; Alt, alteration; P, possibly damaging; T, tolerated; U, unknown; 1000g, 1000 Genomes Project databases (2015version); B, benign; D, deleterious; US, uncertain significance; LB, likely benign; LP, likely pathogenic; P, pathogenic; LQTs, long QT syndrome; Brs, Brugada syndrome. –: no report.

### Conservation analysis and functional districts of the *KCNH2* potassium channel

The site (p.D501) of amino acid of the *KCNH2* protein has different transcripts such as p.D161 and p.D405 (Figure 3). Conservation analyses demonstrate that amino acid sequences are highly conserved in the mutant sites (*KCNH2* p.D501) among various species, including primate, euarchontoglires, laurasiatheria, afrotheria, mammal, birds, sarcopterygii, and fish. According to the summary of a previous study (20), *α* subunit encoded by *KCNH2* involves the N-terminus (NH3^+^), six membrane-spanning segments, and the C-terminus portion (COO^−^). The numbers in the parentheses refer to the position of the amino acid beginning at the N-term position (1th), the beginning of the transmembrane non-pore S1–S4 sequence (398th), the beginning of the transmembrane S5-loop-S6 sequence (552th), the end of the transmembrane S6 sequence (657th), and at the C-term end position (1159th). There are four main prespecified regions: (1)N-terminus, (2)transmembrane “non-pore” region (S1–S4), (3)transmembrane “pore” region (S5-loop-S6), and (4)C-terminus. The *KCNH2* p.D501N mutation is localized to the S3 helix of the transmembrane “non-pore” region (S1–S4).

### Changes in the secondary structure

In the secondary structure prediction analysis, the *KCNH2* p.D501N mutation is predicted to increase the proportion of *α*-helices and extended strands, while reducing the proportion of random coils (Table 2).

**Table 2:** The prediction of the secondary structure, protein properties, and physical properties.

Parameters	WT-*KCNH2*	*KCNH2* p.D501N
The secondary structure predicted by the SOPMA algorithm		
Alpha helix (Hh)	305 (34.35%)	311 (35.02%)*
helix (Gg)	0 (0.00%)	0 (0.00%)
Pi helix (Ii)	0 (0.00%)	0 (0.00%)
Beta bridge (Bb)	0 (0.00%)	0 (0.00%)
Extended strand (Ee)	117 (13.18%)	123 (13.85%)*
Beta turn (Tt)	47 (5.29%)	39 (4.39%)
Bend region (Ss)	0 (0.00%)	0 (0.00%)
Random coil (Cc)	419 (47.18%)	415 (46.73%)*
Ambiguous states	0 (0.00%)	0 (0.00%)
Other states	0 (0.00%)	0 (0.00%)
The protein properties evaluated by the ProtParam tool (ExPASy) algorithm		
Molecular weight	97,541.75	97,540.77*
Theoretical pI	8.75	8.81*
Total number of negatively charged residues (Asp + Glu)	78	77*
Total number of positively charged residues (Arg + Lys)	89	89
Carbon (C)	4,377	4,377
Hydrogen (H)	6,887	6,888*
Nitrogen (N)	1,215	1,216*
Oxygen (O)	1,229	1,228*
Sulfur (S)	42	42
Instability index (II)	44.76	44.76
Aliphatic index	93.96	93.96
Grand average of hydropathicity (GRAVY)	0.036	0.036
Physical properties predicted by the EMBOSS Pepstats algorithm		
Average residue weight	109.844	109.843
Charge	24.5	25.5*
Isoelectric point	8.4742	8.5459*
A280 molar extinction coefficients		
Reduced	116,770	116,770
Cystine bridges	118,020	118,020
A280 extinction coefficients 1 mg/mL		
Reduced	1.197	1.197
Cystine bridges	1.210	1.210
Improbability of expression in inclusion bodies	0.825	0.833*
Tiny (A + C + G + S + T)	285 (32.095%)	285 (32.095%)
Small (A + B + C + D + G + N + P + S + T + V)	461 (51.914%)	461 (51.914%)
Aliphatic (A + I + L + V)	293 (32.995%)	293 (32.995%)
Aromatic (F + H + W + Y)	102 (11.486%)	102 (11.486%)
Non-polar (A + C + F + G + I + L + M + P + V + W + Y)	533 (60.023%)	533 (60.023%)
Polar (D + E + H + K + N + Q + R + S + T + Z)	355 (39.977%)	355 (39.977%)
Charged (B + D + E + H + K + R + Z)	194 (21.847%)	193 (21.734%)*
Basic (H + K + R)	116 (13.063%)	116 (13.063%)
Acidic (B + D + E + Z)	78 (8.784%)	77 (8.671%)*
The hydropathicity predicted by the ProtScale algorithm		
495th amino acid	0.433	0.433
496th amino acid	0.467	0.467
497th amino acid	0.433	0.433
498th amino acid	0.789	0.789
499th amino acid	0.944	0.944
500th amino acid	1.578	1.578
501th amino acid	1.822	1.822
502th amino acid	2.422	2.422
503th amino acid	1.933	1.933
504th amino acid	1.822	1.822
505th amino acid	0.933	0.933
The phosphorylation of proteins predicted by the NetPhos algorithm		
475th amino acid	Unsp (YES)	Unsp (YES)
515th amino acid	CKII, PKA (YES)	CKII, PKA (YES)

* Significant difference.

### Changes in the protein tertiary structure

As presented in Table 3, Missense3D prediction identified three types of key structural damage induced by the *KCNH2* p.D501N mutation, with no other structural abnormalities detected. Specifically, (1) buried charge replacement: The wild-type aspartic acid (Asp501) is a buried charged residue with a relative solvent accessibility (RSA) of 0.0%, while the mutant asparagine (Asn501) is an uncharged residue with an RSA of 8.2%, indicating a substitution of a buried charged residue with an uncharged one. (2) Buried salt bridge breakage: The wild-type Asp501 forms a stable buried salt bridge via its OD1 atom with the NE atom of arginine (Arg534), with an interatomic distance of 3.784 Å (within the threshold of ≤5.0 Å for salt bridge formation). This salt bridge is completely disrupted by the D501N substitution, and the wild-type Asp501 residue has an RSA of 0.0%, confirming that it is a buried salt bridge. (3) Cavity expansion: The substitution leads to a significant expansion of the cavity volume by 458.352 Å^3^, far exceeding the criterion of ≥70 Å^3^ for defining cavity alteration, indicating a marked spatial conformation change in the protein.

**Table 3:** The structural damage induced by *KCNH2* p.D501N, detected by Missense3D prediction.

Parameters	Criterion	Predicting details	Results
Disulfide breakage	The substitution breaks a disulfide bond that was in the wild type. The maximum S-S length for the bond is 3.3 Å.	The wild-type residue is not CYS; so, it cannot form a disulfide bond	−
Buried Pro introduced	The substitution introduces a buried proline.	This substitution does not introduce a proline.	−
Clash	The mutant structure has a MolProbity clash score ≥30, and the increase in the clash score is >18 compared with the wild type.	This substitution does not trigger a clash alert. The local clash score for the wild type is 15.03 and the local clash score for the mutant is 20.73.	−
Buried hydrophilic introduced	The substitution replaces a buried hydrophobic residue with a hydrophilic residue.	This substitution does not replace a buried hydrophobic residue with a hydrophilic residue. The wild-type residue ASP is a buried hydrophilic residue with an RSA of 0.0%, and the mutant residue ASN is a buried hydrophilic residue with an RSA of 8.2%.	−
Buried charge introduced	The substitution replaces a buried uncharged residue with a charged residue.	This substitution does not trigger a buried uncharged residue alert. The wild-type residue ASP is a buried charged residue with a relative solubility of amino acid residues of 0.0%, and the mutant residue ASN is a buried uncharged residue with an RSA of 8.2%.	−
Secondary structure altered	A substitution results in a change in the DSSP secondary structure assignment at the variant position.	His substitution does not alter the secondary structure ‘H’ (4-turn helix).	−
Buried charge switch	Criterion: The substitution switches the charge (+/−) of the buried residue.	This substitution does not trigger a buried charge switch alert. The wild-type residue ASP is buried negative-charged with an RSA of 0.0%, and the mutant residue ASN is buried uncharged with an RSA of 8.2%.	−
Disallowed phi/psi	The mutant residue is in outlier region, while the wild-type residue is in the favored or allowed region.	This substitution does not trigger a disallowed phi/psi alert. The phi/psi angles are in the favored region for the wild-type residue and in the favored region for the mutant residue.	−
Buried charge replaced	The substitution replaces a buried charged residue with an uncharged one.	This substitution replaces a buried charged residue (ASP, RSA 0.0%) with an uncharged one (ASN).	+
Buried Gly replaced	The substitution replaces a buried glycine.	This substitution does not replace a buried GLY residue.	−
Buried H-bond breakage	The substitution breaks all side-chain/side-chain H-bond(s) and/or side-chain/main-chain H-bond(s) formed by the wild type, which were buried. The maximum H-bond N-O length is 3.9 Å.	This substitution does not result in a complete disruption of all side-chain/side-chain H-bond(s) and/or side-chain/main-chain bond(s) bonds formed by a buried ASP residue (RSA 0.0%).	−
Buried salt bridge breakage	The substitution breaks a salt bridge formed by the wild type, which was buried. The maximum N-O bond length is 5.0 Å.	This substitution disrupts a salt bridge formed by the OD1 atom of ASP 501 and the NE atom of ARG 534 (distance: 3.784 Å). The wild-type residue has an RSA of 0.0%.	+
Cavity altered	The substitution leads to an expansion or contraction of the cavity volume of ≥70 Å^3^. The cavity also refers to a pocket on the surface.	The substitution leads to an expansion of cavity volume by 458.352 Å^3^.	+
Buried/exposed switch	The substitution results in a change between the buried and exposed states of the target variant residue. (RSA <9% for buried, and the difference between RSA has to be at least 5%.)	The wild-type residue ASP is buried (RSA 0.0%) and the mutant residue ASN is buried (RSA 8.2%).	−
Cis pro replaced	A cis proline in the wild type is replaced in the mutant.	The wild-type residue is not a cis proline.	−
Gly in a bend	The wild-type residue is glycine and is located in a bend curvature (reported “S” in DSSP)	The wild-type residue is not GLY.	−

Q12809 | 5va2 (A), position: 501 (501 in PDB), variant: ASP > ASN.

Other structural parameters (e.g., disulfide breakage, buried proline introduction, clash, secondary structure alteration, and buried hydrogen bond breakage) showed no abnormalities. The local clash score of the wild-type structure was 15.03, and that of the mutant was 20.73, which did not meet the clash alert criterion (MolProbity clash score ≥30 and increase >18). In addition, the substitution did not induce any of the following structural changes: introduction of buried hydrophilic residues, switching of buried charged residues, adoption of disallowed phi/psi angles, replacement of buried glycine residues, switching between buried and exposed states, replacement of cis proline, or occurrence of glycine within bends. Furthermore, the secondary structure “H” (4-turn helix) at the mutation site remained unchanged.

**Figure 4:**
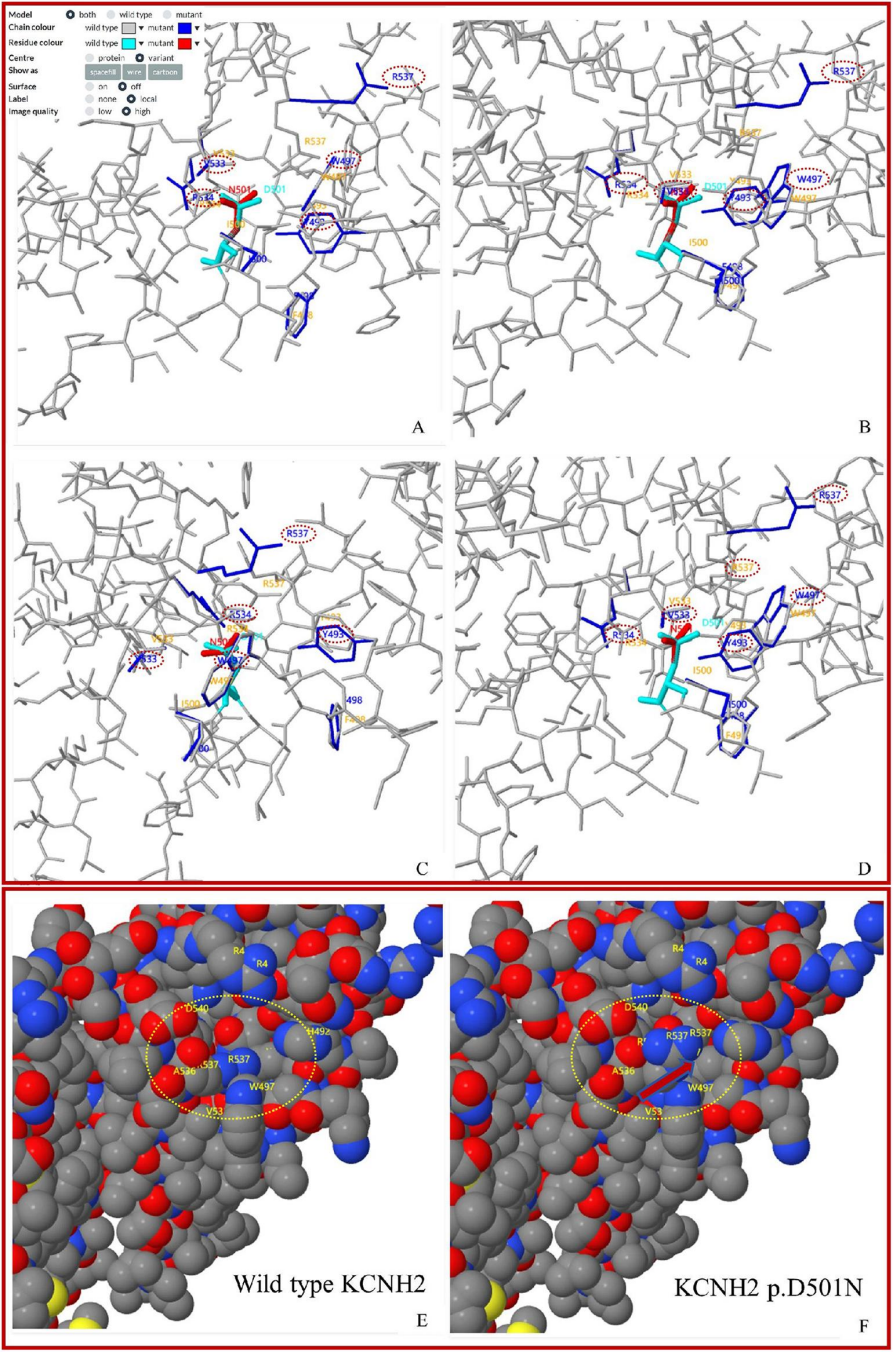
Missense3D-predicted structural changes induced by the *KCNH2* p.D501N mutation. This figure illustrates the tertiary structure abnormalities of the K_v_11.1 protein caused by the *KCNH2* p.D501N mutation, as predicted by Missense3D (based on PDB ID: 5VAO, the experimental 3D structure of the K_v_11.1 voltage-sensing domain). **(A–D)** Evaluation of the impact on the amino acid spatial structure between wild-type *KCNH2* (D501 in blue font) and mutant *KCNH2* p.D501N (N501 in red font) from different spatial angles. In the same figure, we simultaneously present the spatial structure of amino acids around wild-type D501 and mutant N501, as well as the spatial structural changes induced by the mutation, and observe and assess the impact of the mutation on the surrounding amino acid space from multiple perspectives. The wild-type *KCNH2* (D501) is a buried negatively charged residue that forms a stable salt bridge with Arg534 (R534), and its spatial structure is displayed in yellow font and gray amino acid graphics. The *KCNH2* p.D501N mutation replaces the buried charged aspartic acid (D501) with uncharged asparagine (N501), resulting in buried charge replacement and breakage of the D501-R534 salt bridge. The induced spatial structural changes in the amino acids are shown in blue font and blue amino acid graphics, among which amino acids with obvious spatial displacement (Arg537, Arg534, Tyr493, Val533, and Trp497) are highlighted with red dashed ellipses; these changes disrupt the intermolecular interactions among these residues and cause spatial structure deflection. **(E,F)** Cavity volume change: The wild-type **(E)** has a compact cavity (yellow area) near D501; the mutant **(F)** leads to a 458.352 Å^3^ expansion of the cavity (yellow area), with R537 moving markedly outward (red arrow) from the original motif, which is a key manifestation of the structural abnormality induced by the mutation. Collectively, these changes, including buried charge replacement, D501-R534 salt bridge breakage, cavity expansion, and disrupted residue interactions, impair the function of the voltage sensor domain (VSD), thereby contributing to the pathogenesis of long QT syndrome Type 2 (LQT2).

As illustrated in the optimized Figure 4, *KCNH2* p.D501N disrupts intermolecular interactions among residues Arg537 (R537), Arg534 (R534), Tyr493 (Y493), Val533 (V533), and Trp497 (W497), leading to spatial structural displacement and deflection, with the most prominent changes observed in R537, R534, and Y493. The mutant also causes a marked outward displacement of the R537 residue from the motif, which contributes to cavity expansion.

### The changes in protein physical–chemical properties

Compared with the wild-type *KCNH2* protein (Table 2), the *KCNH2* p.D501N protein decreased the molecular weight, the total number of negatively charged residues (Arg + Lys), the amount of oxygen, charged residues (B: Asx, D: Asp, E: Glu, H: His, K: Lys, R: Arg, and Z: Glx), and the acidic (B + D + E + Z) but increased the theoretical PI, the amounts of hydrogen and nitrogen, the isoelectric point, the charge, and the improbability of expression in inclusion bodies. The *KCNH2* p.D501N protein did not change the hydropathicity and phosphorylation of the protein properties, unlike the wild-type *KCNH2* protein.

## Discussion

In this study, we identified the *de novo KCNH2* p.D501N mutation in a 16-year-old girl with early-onset malignant LQT2, characterized by a QT interval ≥600 ms, recurrent TdP, VT, and VF that required ICD implantation. Our findings demonstrate that *KCNH2* p.D501N is predicted to not only induce abnormal changes in the secondary structure of the K_v_11.1 protein (increased *α*-helices and extended strands, decreased random coil) but also cause tertiary structure abnormalities such as buried charge replacement, breakage of the D501-R534 salt bridge, cavity expansion (with a marked outward displacement of R537), and disruption of S3–S4 helix interactions. These structural alterations may further modify the physicochemical properties of K_v_11.1, ultimately impairing the rapid delayed rectifier potassium current (I_Kr_) and leading to malignant LQT2.

The *KCNH2* p.D501N mutation was first identified as a pathogenic mutation in a patient with congenital LQTS (lack of detailed clinical data) in 2002 and an 11-year-old girl with a QT interval of 490 ms (21, 22). In 2009, *KCNH2* p.D501N was also detected in a 5-year-old boy with LQT2 complicated by an isolated non-compaction of the ventricular myocardium (23). Compared with these previous reports, this study has obvious supplementary value. First, this study clarified the structural changes in the K_v_11.1 protein that were induced by *KCNH2* p.D501N, including buried charge replacement, breakage of the D501-R534 buried salt bridge, 458.352 Å³ cavity expansion, and spatial displacement of the R537 residue. Second, this study clarified its penetrance as 38%, with CADD > 20, PROVEAN = −4.633, and REVEL = 0.915, providing a quantitative basis for clinical genetic counseling. Last, this study is the first to report the early-onset malignant phenotype of this *de novo* mutation in Chinese adolescents without myocardial structural abnormalities, thus expanding the clinical phenotypic spectrum of this mutation.

Loss-of-function mutations in *KCNH2* are the leading cause of LQT2 (24). The human *KCNH2* gene encodes the pore-forming subunit of the K_v_11.1 voltage–gated potassium channel, which mediates the I_Kr_ current critical for ventricular repolarization. Structurally, K_v_11.1 comprises six transmembrane segments (S1–S6), with S1–S4 forming the VSD and S5–S6 (along with the intervening pore loop) constituting the pore domain (25, 26). The VSD undergoes voltage-dependent outward displacement in response to transmembrane electric field changes, which is electromechanically coupled to the activation gate of the K_v_11.1 channel. This structural rearrangement is transmitted to the pore domain via physical interactions between the S4–S5 linker and the cytoplasmic terminal of the S6 helix, regulating channel gating (26). The S4 domain contains positively charged residues (Lys525, Arg528, Arg531, Arg534, and Arg537) that sense transmembrane electrical field changes, and neutralization of any of these residues can alter the voltage dependence of activation gating (26). Notably, a network of internal salt bridges between positively charged residues in S4 and negative charges in the S1–S3 transmembrane helices modulates VSD movement (27); for example, Lys525-Asp411 and Arg531-Asp460/Asp509 form potential salt bridges in the closed and open states of the K_v_11.1 channel, respectively (27, 28).

*KCNH2* p.D501 is localized to the intracellular half of the S3 helix in the VSD and is highly conserved across species. Notably, this residue is conserved across different *KCNH2* transcripts (e.g., D161 in the short transcript, D405 in the intermediate transcript), emphasizing its critical functional role. As a negatively charged residue, D501 contributes significantly to voltage sensing during K_v_11.1 activation by forming electrostatic interactions with positively charged residues (e.g., Arg534) in the S4 helix (27, 29). This interaction stabilizes the VSD structure and ensures efficient voltage-dependent gating. The p.D501N mutation replaces the negatively charged aspartic acid with uncharged asparagine, disrupting the D501-R534 salt bridge (interatomic distance: 3.784 Å in the wild type) and inducing a 458.352 Å³ cavity expansion caused by an outward displacement of R537. These changes impair the electrostatic coupling between the S3 and S4 helices, reducing the voltage sensitivity of channel activation (as reflected by a decreased gating charge z_a_ in homologous mutations (29) and destabilizing the open state of the channel. Consequently, the K_v_11.1 channel enters the inactivated state more rapidly, altering ion flux through the channel, prolonging the duration of the ventricular myocyte action potential, and increasing the transmural dispersion of repolarization, thereby creating an electrophysiological substrate for TdP, VT, and VF, which is consistent with the severe clinical phenotype of the proband.

A homologous mutation at this site, *KCNH2* p.D501C, has been shown to exert multifaceted effects on K_v_11.1 function. *In vitro* experiments have confirmed that p.D501C induces a depolarizing shift in the channel activation curve, increases the opening rate constant at specific voltages, accelerates inactivation and deactivation, and retains high potassium ion selectivity, ultimately leading to QT interval prolongation in patients (29). Notably, the pathogenic mechanism of the p.D501N mutation predicted by bioinformatics in this study (neutralization of D501 negative charge, VSD function abnormality) is highly consistent with the *in vitro* experimental results of p.D501C—both neutralize the negative charge of D501, impair electrostatic interactions with S4 residues, induce abnormal channel gating, and further lead to abnormal I_Kr_ current and QT interval prolongation. The *in vitro* experimental evidence of this homologous mutation indirectly supports the reliability of the bioinformatics prediction results of this study. However, our study extends these findings by demonstrating that p.D501N specifically induces salt bridge breakage, cavity expansion, and altered residue interactions (R537, R534, Y493, V533, and W497), providing novel insights into the structural basis of pathogenicity for mutations at this site.

Notably, the S3 helix of the K_v_11.1 VSD and adjacent S1–S2 helices form a conserved “voltage-sensing charge network” (26, 29), where other mutations are likely to induce LQT2 through the same mechanism. For instance, both the p.D509C mutation (S3 helix) and the p.D466C mutation (S2 helix) disrupt the electrostatic balance between VSD helices (29), leading to depolarizing shifts in the activation curve and accelerated deactivation-phenotypic changes consistent with those induced by p.D501N and p.D501C. In addition, the structural abnormalities related to the *KCNH2* p.D501N mutation identified by the bioinformatics analysis in this study (buried salt bridge breakage, cavity expansion, R537 residue displacement, etc.) not only improve the pathogenic mechanism of the mutation but also provide clear research targets for subsequent *in vitro* and *in vivo* mechanistic experiments. Our research also indicates that mutations targeting conserved charged residues in the S1-S3 helices of the VSD share a common pathogenic pathway: “disruption of the voltage-sensing charge network → VSD dysfunction → I_Kr_ reduction → ventricular repolarization delay.” This conserved network explains why mutations targeting other charged residues in S1–S3 helices (e.g., p.D509C and p.D466C) share similar pathogenic phenotypes, highlighting a universal regulatory role of electrostatic balance in VSD function. This finding highlights the strong genotype–phenotype correlation of the VSD in LQT2, providing a theoretical basis for pathogenic prediction of related mutations.

Clinically, unlike previous studies that only identified the mutation, our study provides long-term clinical follow-up data for up to 11 years (2015–2026) for the first time, confirming the long-term efficacy of ICD combined with *β*-blocker (metoprolol succinate sustained-release tablets) and potassium–magnesium supplementation (potassium aspartate and magnesium aspartate tablets) in the management of malignant LQT2 caused by a VSD-related *KCNH2* p.D501N mutation. The patient's syncope symptoms were completely relieved, the frequency of TdP/VT episodes was significantly reduced, with only 2 ICD discharges recorded in 2024, and no adverse drug reactions occurred. These follow-up data provide a solid empirical basis for the long-term clinical management of such patients. Metoprolol, as a β1-selective blocker, targets the mutation-induced sympathetic sensitivity of the K_v_11.1 channel: the p.D501N mutation disrupts the electrostatic coupling between the S3 and S4 helices of the VSD, making the channel more susceptible to catecholamine-induced gating abnormalities (26). Metoprolol inhibits sympathetic activation, reduces the phosphorylation of K_v_11.1 channels, stabilizes the structural conformation of the mutant VSD, and decreases the transmural dispersion of repolarization, thus lowering the risk of TdP/VT (30). In addition, potassium and magnesium ions are essential for maintaining normal I_Kr_ current function: the mutant K_v_11.1 channel has reduced K^+^ conductance due to disrupted VSD-pore domain coupling, and supplementation with potassium aspartate and magnesium aspartate helps maintain the intracellular K^+^ gradient, partially compensating for the loss of function of the mutant channel and stabilizing ventricular repolarization (31).

## Limitations

A limitation of this study is its exclusive focus on a single known pathogenic mutation (KCNH2 p.D501N). The findings could be further strengthened by expanding the clinical cohort and providing additional mechanistic details at the molecular level. The functional analysis of the mutation is limited to in silico bioinformatics prediction, and the results of the study need to be verified by *in vitro* experiments and *in vivo* animal experiments. In addition, the main limitation of this study is that it is a single case report with a small sample size, and the universality of the research conclusions needs to be further verified. To address the issues of the present inadequate mechanistic verification and small sample size, a series of research studies need to be conducted in the future. On the one hand, the plan for mechanistic experiment verification needs to be implemented, *in vitro* patch-clamp technology must be used to detect changes in I_Kr_ current, Western blot must be used to detect the expression and cellular localization of the K_v_11.1 protein, and immunofluorescence technology must be employed to observe VSD structural changes, further verifying the bioinformatics prediction results and providing more direct experimental evidence for the pathogenic mechanism of the mutation. On the other hand, based on the research findings of this single case, the sample size must be expanded, multicenter, prospective studies must be conducted to verify the universality of the structural abnormalities of the mutation, the quantitative pathogenicity indices and treatment plans devised in this study must be studied further, and the clinical phenotypic spectrum of the mutation must be expanded further. At the same time, combined with the results of mechanistic experiments, future studies need to deeply explore the pathogenic mechanism of the mutation, provide a more solid theoretical and clinical basis for the accurate diagnosis, risk stratification, and individualized treatment of LQTS, reflect the true scientific research value of this single case report, and enhance its long-term impact.

## Conclusion

The *de novo KCNH2* p.D501N first reported in the Chinese family discussed in this study is predicted to not only change the secondary structure of the K_v_11.1 protein but also induce tertiary structure abnormality, including buried charge replacement, buried salt bridge (formed by p.D501 and p.R534) breakage and cavity expansion (p.R537 markedly moving outward), and disruption of S3 and S4 helix interaction of the VSD, all of which may potentially influence the physical–chemical properties of the protein and the subsequent function of I_Kr_ current, eventually leading to early-onset and malignant LQT2. It can be concluded that this study provides insights into the structural and physicochemical basis of the pathogenicity of the VSD-related *KCNH2* p.D501N mutation and confirms the efficacy of the *β*-blocker combined with ICD therapy for this patient population.

## Data Availability

The datasets presented in this study are available in online public repositories. The names of the repositories and accession number(s) can be found in the article and Supplementary Material. The detailed individual patient data involved in this study are also available from the first or corresponding author upon reasonable request and with approval from the relevant Chinese authorities.
